# Supplementary material for: Quality and sustainability of Ethiopia’s national surgical indicators
Source: PLOS Glob Public Health. 2024 Mar 27;4(3):e0002600. doi: 10.1371/journal.pgph.0002600 (PMC10971685; doi:10.1371/journal.pgph.0002600)
Supplement: Attachment — Submitted filename: Response to Reviewers.docx [file pgph.0002600.s003.docx]

Dear Dr. Alayande and the Editorial Team of PLOS Global Public Health,

Thank you very much for the review of our manuscript and for your specific feedback. Below are our comments for each point.

- Kindly add in more detail on the ethics approval/Institutional Review Board documentation into the text. Adding the numbers on the ethics approval/certificate makes for a more transparent report. Kindly provide these details for the Medical College of Wisconsin and the Ethiopian Public Health Institute.
  - **This has been updated with the study numbers.**
- Also add in a clearer data availability statement, making limitations to public data sharing clear in the Data Availability Statement at the time of submission. "PLOS journals require authors to make all data necessary to replicate their study’s findings publicly available without restriction at the time of publication. When specific legal or ethical restrictions prohibit public sharing of a data set, authors must indicate how others may obtain access to the data. This data refers to 'the minimal dataset' required to replicate all study findings reported in the article, as well as related metadata and methods." The latter seems to be the case with this work, but the data availability statement will be seen by all, including the fact that it should not be published. PLOS recognizes that, *in some instances, authors may not be able to make their underlying data set publicly available for legal or ethical reasons*. This data policy does not overrule local regulations, legislation or ethical frameworks. *Where these frameworks prevent or limit data release, authors must make these limitations clear in the Data Availability Statement at the time of submission*. Acceptable restrictions on public data sharing are detailed on the PLOS site. Also note it is not acceptable for an author to be the sole named individual responsible for ensuring data access.
  - This has been updated to read:
  - **“PLOS journals require authors to make all data necessary to replicate their study’s findings publicly available without restriction at the time of publication. When specific legal or ethical restrictions prohibit public sharing of a data set, authors must indicate how others may obtain access to the data. This data refers to 'the minimal dataset' required to replicate all study findings reported in the article, as well as related metadata and methods. For this study, anonymized data for review has been uploaded as a supporting information file titled "Data repository." This data was shared with the study investigators with the permission of the Ethiopian Federal Ministry of Health, the relevant Regional Health Bureaus for the regions described, and the participating hospitals. The names of the specific hospitals have been removed for confidentiality purposes in compliance with ethics review boards. The Ethiopian Federal Ministry of Health retains the rights to this data and therefore prevents additional release of data, beyond what is provided in the supporting information file.”**
- Please include a complete copy of PLOS’ questionnaire on inclusivity in global research in your revised manuscript. Our policy for research in this area aims to improve transparency in the reporting of research performed outside of researchers’ own country or community. The policy applies to researchers who have travelled to a different country to conduct research, research with Indigenous populations or their lands, and research on cultural artefacts. The questionnaire can also be requested at the journal’s discretion for any other submissions, even if these conditions are not met.  Please find more information on the policy and a link to download a blank copy of the questionnaire here: [https://journals.plos.org/globalpublichealth/s/best-practices-in-research-reporting](https://urldefense.com/v3/__https:/journals.plos.org/globalpublichealth/s/best-practices-in-research-reporting__;!!H8mHWRdzp34!42p3eJreM2notPrH6CjlnHgQEAEhFoCG9V_ign8GyuIguhaPdFIPNYBp1_Ns8kQorj4pxQ62I_juCBE$). Please upload a completed version of your questionnaire as Supporting Information when you resubmit your manuscript.
  - **Thank you, this has been answered and included.**
- Please amend your online Financial Disclosure statement. If you did not receive any funding for this study, please simply state: “The authors received no specific funding for this work.”
  - **This has been completed and now reads “Study author Cook KR received travel funding through the Dr. Elaine Kohler Summer Academy of Global Health Research Scholarship, funded by the Wm. Collins Kohler Foundation. This funder had no role in study design, data collection and analysis, decision to publish, or preparation of the manuscript. The other author(s) received no specific funding for this work.”**
- Please update your online Competing Interests statement. If you have no competing interests to declare, please state: “The authors have declared that no competing interests exist.”
  - **This has been completed.**
- Some material included in your submission may be copyrighted. According to PLOS’s copyright policy, authors who use figures or other material (e.g., graphics, clipart, maps) from another author or copyright holder must demonstrate or obtain permission to publish this material under the Creative Commons Attribution 4.0 International (CC BY 4.0) License used by PLOS journals. Please closely review the details of PLOS’s copyright requirements here: PLOS Licenses and Copyright. If you need to request permissions from a copyright holder, you may use PLOS's Copyright Content Permission form. Please respond directly to this email or email the journal office and provide any known details concerning your material's license terms and permissions required for reuse, even if you have not yet obtained copyright permissions or are unsure of your material's copyright compatibility.
  - Figure 1: Please confirm whether you drew the images / clip-art within the figure panels by hand. If you did not draw the images, please provide (a) a link to the source of the images or icons and their license / terms of use; or (b) written permission from the copyright holder to publish the images or icons under our CC-BY 4.0 license. Alternatively, you may replace the images with open source alternatives. See these open source resources you may use to replace images / clip-art:
    - [https://commons.wikimedia.org](https://urldefense.com/v3/__https:/commons.wikimedia.org__;!!H8mHWRdzp34!42p3eJreM2notPrH6CjlnHgQEAEhFoCG9V_ign8GyuIguhaPdFIPNYBp1_Ns8kQorj4pxQ62rsRwihE$)
    - [https://openclipart.org/](https://urldefense.com/v3/__https:/openclipart.org/__;!!H8mHWRdzp34!42p3eJreM2notPrH6CjlnHgQEAEhFoCG9V_ign8GyuIguhaPdFIPNYBp1_Ns8kQorj4pxQ62OdStm2Q$)
  - **Regarding Figure 1, the study authors have emailed the copyright policy of Canva, the source of the icons, to ensure copyright compatibility.**
- Please review your reference list to ensure that it is complete and correct. If you have cited papers that have been retracted, please include the rationale for doing so in the manuscript text, or remove these references and replace them with relevant current references. Any changes to the reference list should be mentioned in the rebuttal letter that accompanies your revised manuscript. If you need to cite a retracted article, indicate the article’s retracted status in the References list and also include a citation and full reference for the retraction notice.
  - **The references have been reviewed and are accurate.**

Please address all the reviewer's concerns concerning the limitations and recommendations. This is required for acceptance. Both reviewers of this work are very knowledgeable about the local context and have suggested thoughts around the recommendations and improving the limitations section. None of the concerns were major, but applying these thoughts can help improve the draft, and clarify concerns that readers might raise. Overall, this is very clear and well done.

**Comments to the Author**

5. Review Comments to the Author

Reviewer #1: Based on the Saving Lives through Safe Surgery (SaLTS) initiative launched by the Ethiopian Federal Ministry of Health, introducing 15 key performance indicators (KPIs) to standardize data practices, the study aims to evaluate KPI data collection practices and assess data quality across 10 hospitals in two regions. The investigation compares five selected key indicators, including Surgical Volume, Perioperative Mortality Rate (POMR), Adverse Anesthetic Outcome (AAO), Surgical Site Infection (SSI), and Safe Surgery Checklist (SSC) Utilization. Findings reveal discrepancies in data consistency, with incomplete information in registries and challenges related to non-standardized reporting forms. Facilitators include the continued use of registries, while barriers involve variable indicator recording and delayed indicator rollout. Recommendations focus on continuous quality checks and capacity building to enhance data management for improved healthcare systems.

Overall, the study is relevant, and well written with specific comments and minor adjustment suggestions described below for the authors' consideration.

**Thank you very much to Reviewer 1 for your accurate summary of the manuscript and careful review of the study. We are happy to provide responses and edits based on your comments below.**

Introduction: It provides a good overview of the global significance of surgical care data and connects it well into available data within the country.

Methods: The study design, data analysis, and method of measure are articulated clearly. The rationale with selection of study sites is well explained, however, information on the reason behind the chosen time frame (Jan-June 2022) should be further clarified and justified.

**The rationale for the time period was to review six months of past data at each hospital from the time of the inquiry, using the period of Jan-June 2022 as the most recent six months of hospital data available. We did not do a longer period of time (1 year or more) based on time and resource constraints. This has been updated under the Study Design section of the Materials and Methods: “This time period was chosen to evaluate the most recent six-months of hospital data from the time of inquiry.”**

Results: The main findings are presented clearly with effective summarization through appropriate table and figures.

Discussion: It provides insightful points, drawing comparison with findings from similar studies done in other countries.

Limitations: The study offers a comprehensive overview of its limitations. However, it would be beneficial to further elaborate on any countermeasures implemented by the study team to mitigate potential biases encountered, if any.

**Thank you for this comment. The main potential biases encountered are related to the hospital and region selection. This selection was based on prior studies, those involved in Safe Surgery 2020 initiatives, and logistic feasibility. We have included more explanation and detail about this in the first paragraph of the limitations.**

**“National implications of this study are limited by the small sample size (ten hospitals) and limited study period (six months). Hospital selection was also limited to facilities with prior involvement in Safe Surgery 2020. This limitation could potentially impact the generalizability of these findings to the current surgical landscape in Ethiopia, as system changes are ongoing and interregional differences are likely. The investigators included two regions within Ethiopia as well as both primary and general hospitals as countermeasures to the limited sample size and period. Selecting hospitals involved in the prior surgical data strengthening program (in Amhara) and those not (in Sidama) was also employed to expose potential differences in data practices.”**

Recommendations: While the need for qualitative assessment was acknowledged as a limitation in the study, incorporating a recommendation for future studies to explore the reasons behind data discrepancies would significantly contribute to devising targeted interventions for improving the quality of surgical care data.

**We have made this more explicit in the Limitations section at the end of the first paragraph. Qualitative studies with the local data managers are underway and we anticipate these results to be forthcoming shortly. We agree that this is an essential aspect for improvement of the data system.**

**“Follow-up investigation using qualitative methods, specifically surveying data collectors and data managers at participating hospitals and data regions, will provide further context around the findings. Future studies are also planned to expand this initial investigation of key performance indicator data in Ethiopia to a larger number of hospitals and regions.”**

Conclusion: Major points and messages are effectively summarized.

In summary, the study provides valuable insights into KPI data collection practices within the SaLTS initiative underscoring the need for continuous quality improvement efforts to ensure a robust surgical database in the country and provides a strong foundation for future research.

**Thank you again for your review and comments which have strengthened the manuscript.**

Reviewer #2: I have carefully reviewed this scientific paper on the quality and sustainability of Ethiopia’s national surgical care indicators.

**We appreciate the very thoughtful comments and close reading of the manuscript. Below we have responded to the explicit inquiries and recommendations below.**

1. Relevance and Significance- The paper effectively addresses a critical area of concern in surgical care in LMICs particularly assessing the nuance of surgical indicators in terms of sustainability integrity, gaps, and areas of potential improvement. The relevance of the topic is evident given the global movement and effort to improve access to quality, safe, timely, and appropriate surgical care and the need for data driven decision making.

2. Methodology- The methodology section was well detailed, providing data collection, analysis, definitions of framework, completeness etc. Key concerns re: data integrity were also raised although I recommend a more robust discussion of the limitations (which authors have done later in the limitation section).

**Thank you for this comment. We have significantly edited the Limitations section of the manuscript to better address some of the shortcomings regarding data integrity and data quality with a more thorough discussion. We hope this addresses your concerns.**

3. Data Presentation and Analysis- the data presentation's is generally clear and analysis is appropriately conducted. Some visual aids such as tables and charts were used which is good. However, a more detailed graph or analysis in writing which tries to break down the key points within the table would make the information more absorbable.

**We appreciate this suggestion. Table 3 includes the main results from the study, in a format providing more detailed numbers to summarize the 6 months of data. Whereas Figure 2 provides an overall impression of the level of discrepancy between the primary hospital data and national data for each facility in graphic form. The main interpretation related to the data discrepancies highlighted in Table 3 and Figure 2 are outlined in the last paragraph of the Results under “Data quality”. In this last paragraph we outline the areas where there are complications recorded in the registry data which does not make it into the national DHIS2 data. We have added more explanation around Table 3 to drive home some of these key points in this section and highlight these areas of discrepancy. Additionally, the Discussion has been expanded to enrich this conversation.**

Even further, I would have also loved to see a more robust explanation of the relationship between the metrics, KPI and/or indices and what that entails, both for the paper and for the overall quality of care and in terms of addressing the desired outcome as well as how that impacts the analysis and the overall service delivery and patient care (even if it gets elaborated in the discussion segment).

**This is a very important point. For the relationship between the key performance indicators as a reflection of service delivery and quality of care, we have added some additional interpretation in the Discussion.**

**“An accurate assessment of the surgical system is dependent on these indicators as metrics of service delivery and quality of care. As the surgical system in Ethiopia continues to grow, it is imperative the quality of data is a true reflection of hospital realities. Given these rates of surgical complications may be lower than expected, a more accurate system for these indicators will allow us to track improvements in access to and quality of surgical care.”**

They can indeed try a more sophisticated analysis on Excel which is what they have tried but I generally recommend trying Tableu as a great data visualization tool as it gives the non expert reader a more digestible, high quality, and summarized outcome. This will also hopefully aid high level stakeholders such as decision makers at FMOH an efficient way to aid future policy making and advocacy efforts.

**Thank you for this tip and we will definitely keep this in mind for future graphs and figures! Figure 1 was created in Canva to outline the current data flow mechanism. We believe this graphic provides a reasonable summary of the system in its current format. Figure 2 was created in Excel to show a graphic representation of the number of hospitals with inconsistent or incomplete data for each of the main indicators. We are hoping this can also be used as a quick snapshot of major study findings. We have previously provided a report summarizing in more detail the findings to the FMOH and regional and local stakeholders, which we are hopeful will also be used for future policy decisions and efforts.**

4. Discussions and implications-

A) Standardization of data collection practices: The authors rightly highlight the uniformity in data collection mechanisms but note discrepancies due to non-standardized data collection forms and practices. To address this, it is crucial to recommend the development and implementation of standardized data collection tools across all hospitals. Emphasize the importance of a collaborative effort involving healthcare stakeholders, policymakers, and professional organizations to establish and endorse standardized forms, ensuring consistency in recording and reporting across facilities.

**-The study authors appreciate the recommendation above and have included additional sentences in the Discussion to emphasize the point described:**

**“While standardized registries have improved the collection of data in the setting of their origination, the development and implementation of standardized monthly KPI reporting forms across hospitals would improve consistency in recording and reporting. This will require a collaborative effort between policymakers, healthcare stakeholders, hospital employees, and non-governmental organizations.”**

B. Data Quality and Completeness: While the study finds data completeness and consistency above 70% for the five KPIs, making recommending specific measures to enhance data quality would . This making granular suggestions and recommendations may enhance their work. This could be regular training sessions for healthcare staff involved in data collection, routine data quality checks, and the utilization of technology for real-time data entry to minimize errors. Maybe even propose the establishment of a centralized oversight body responsible for monitoring and ensuring the quality and completeness of surgical data at the national level. Similarly, for the below points

C) Addressing inconsistencies in surgical volume and complications

D) Training and retention of quality improvement officers

E) External audits and continuous quality inspections

F) Recommendation for policy makers

**Thank you for this great insight. We have expanded the Recommendations section based on this suggestion to more clearly address each of these points. We really appreciate the additional suggestions for actionable items. Including these in the Recommendations section will allow clearer access for key stakeholders and policy makers. We have split up this into sections related to the national, regional, and hospital level.**

In conclusion, the authors' identification of gaps in surgical data practices is commendable, and the discussion section can be strengthened by providing nuanced and actionable recommendations for addressing these challenges. The proposed enhancements aim to guide policymakers and healthcare stakeholders in implementing practical measures to improve the overall quality and reliability of surgical data in Ethiopia.

**We appreciate this invaluable recommendation and have strengthened the manuscript to address these critiques.**

5. Limitations

A. Sample Size and Study Period: The acknowledgement of the small sample size (10 hospitals) and the limited study period (six months) is appropriate. However, suggest expanding on the potential impact of these limitations on the generalizability of the findings. Discuss whether the findings can be considered representative of the broader healthcare landscape in Ethiopia.

**-The study authors have reviewed the comments above and incorporated the recommendations. Please see the updated manuscript for added sentences concerning this limitation and its effect on the generalizability of the findings. Additionally, the Discussion does relate the findings to other relevant studies in Ethiopia and the region. This allows for the reader to see the comparability of the statistical results to other similar research findings in the area. See first paragraph under Limitations for more discussion on this.**

B. Data Source and Quality: The mention of using registry data as the primary source is well-noted. Suggest discussing the potential biases introduced by relying solely on registry data, such as the underestimation of data quality issues. Encourage a deeper exploration of alternative data sources, like mortality audits, to supplement and validate the findings.

**- The study authors have reviewed the comments above and incorporated the recommendations. Please see the updated manuscript for added sentences concerning this potential bias and the use of mortality audits in future studies to supplement the manuscript’s findings. The study investigators ultimately used registry data as the primary data source, as it represents the first step of the data collection process, the study's key interest.**

C. Reporting Period Variability: The acknowledgment of reporting period variability is crucial. Elaborate on how this variability might affect the accuracy and comparability of the data. Provide recommendations on standardizing reporting periods across hospitals to enhance the consistency of data collection and reporting.

**-The study authors have reviewed the comments above and incorporated the recommendations. Please see the updated manuscript for added sentences concerning this limitation and its effect on the accuracy and comparability of the data, including specific recommendations on standardizing reporting periods across hospitals to reduce this limitation in the future.**

D. Data Correction and Manipulation: Address the limitations associated with the inability to quantitatively assess the extent of data correction and manipulation by hospital staff. Discuss the potential implications of data correction on the reliability of the reported indicators and suggest strategies for minimizing such corrections, ensuring data accuracy.

**-The study authors have reviewed the comments above and incorporated the recommendations – this is more clearly addressed in the Limitations now after further editing. Please see the updated manuscript for added sentences concerning this limitation, its implications, and recommendations for countermeasures to this issue.**

E. Blank Entries and Data Completeness: Discuss the challenges posed by the use of blank entries to denote "0" findings within data forms. Provide recommendations for improving data completeness and suggest alternative methods for capturing zero values without compromising the ability to assess data completeness accurately.

**-The study authors have reviewed the comments above and incorporated the recommendations. Please see the updated manuscript for added sentences concerning this limitation and the challenges it poses, including specific recommendations on improving data completeness and methods for correctly capturing zero values or no recordable events during the reporting period.**

F. Exclusion of Hospitals in Tigray: The exclusion of hospitals in Tigray due to ongoing conflict and travel restrictions is a significant limitation. Discuss the potential impact of this exclusion on the comprehensiveness of the study and suggest strategies for addressing data gaps in conflict-affected regions.

**-The study authors have reviewed the comments above and incorporated the recommendations. Please see the updated manuscript for added sentences concerning this limitation and its impact on the study’s comprehensiveness, including the longitudinal inclusion of Amhara hospitals.**

6. Recommendations

Supervision Mechanism and Data Consistency:

Expand on the recommendation for a regular supervision mechanism to address data inconsistencies. Provide details on the structure and frequency of such supervision, emphasizing its role in enhancing data quality. Consider discussing the potential challenges in implementing and sustaining this mechanism.

**We have further outlined each section of the Recommendations to detail how these supervision systems currently work and could be improved at the national, regional, and hospital level. We have included specific examples where relevant.**

National Emphasis on Data Quality:

Strengthen the recommendation for a national emphasis on data quality. Discuss specific strategies for promoting a culture of data quality at the national level, considering the unique challenges faced in a resource-limited setting. Highlight the importance of balancing the pursuit of high-quality data with avoiding punitive measures for suboptimal outcomes.

**Thank you for this suggestion. We have expanded the Recommendations section to include more specific items that may help improve data quality at the national level. This includes national recognition for high quality data, strengthening the M&E arm of SaLTS, and a uniform electronic health system.**

Regional Survey Teams:

Further elaborate on the recommendation for implementing permanent survey teams at the regional level. Discuss the anticipated impact of such teams on reducing errors and improving data quality. Provide examples of successful implementation in regions where this system is already in place.

**This has been more explicitly defined in the Recommendations section under the paragraph on regional teams. Regional teams currently focus on data completeness as the main quality metric (to ensure DHIS2 data is complete at monthly intervals).** **However, to truly improve data capture, there must be increased focus on consistency and accuracy. We suggest quarterly reviews at the hospital level for this purpose.**

Hospital-Level Quality Improvement Programs:
Enhance the recommendation for more consistent quality improvement programs at the hospital level. Discuss the potential benefits of these programs in fostering better teamwork and increasing data capture. Provide practical suggestions for incorporating these programs into routine hospital practices.

**We have included more specifics related to hospital level QI programs based on the authors’ experience at local hospitals who have successfully implemented such programs. Overall, we think a shift in focus to improving quality of care will help in improving the quality of data.**

Conclusion:

Challenges in Surgical Data Collection:

The conclusion effectively highlights the persistent challenges in accurate surgical data collection in a resource-limited setting. Consider expanding on specific challenges faced by healthcare professionals and institutions, providing context for readers unfamiliar with the intricacies of data collection in such settings.

**This has been expanded.**

Impact of National System:

Emphasize the impact of the national system for data collection and reporting, highlighting its importance to ongoing progress. Discuss the successes and shortcomings of the existing system, acknowledging its role in laying the foundation for future improvements.

**This has been more explicitly emphasized.**

Unified Mechanisms and Continuing Improvement:

Reinforce the need for more unified mechanisms for data transfer, ongoing training, and regular audits to improve the current data structure. Elaborate on how these recommendations align with the broader goals of Ethiopia's national surgical and anesthesia strategic plan.

**This has been addressed in more detail in the Recommendations section.**

Future Developments:

Strengthen the conclusion by discussing future developments, such as the ongoing development of an electronic registry. Explore how these advancements could contribute to addressing current challenges and fostering continuous improvement in tracking surgical capacity and quality in Ethiopia.

**We have added this additional suggestion.**

In summary, while the limitations and recommendations provided are insightful, further elaboration on the potential implications of these limitations and more specific details in the recommendations would strengthen the overall depth and nuance of the discussion. Additionally, enhancing the conclusion with a forward-looking perspective on future developments could provide a more comprehensive and impactful closure to the study.

**We appreciate the detailed comments on the Conclusion. Given the Limitations and Recommendations sections have been significantly expanded to address the above listed issues, we are inclined to keep the Conclusion a bit more concise with one paragraph to reiterate the key points from the study.**

6. PLOS authors have the option to publish the peer review history of their article ([what does this mean?](https://urldefense.com/v3/__https:/journals.plos.org/globalpublichealth/s/editorial-and-peer-review-process*loc-peer-review-history__;Iw!!H8mHWRdzp34!42p3eJreM2notPrH6CjlnHgQEAEhFoCG9V_ign8GyuIguhaPdFIPNYBp1_Ns8kQorj4pxQ62Hxy-tIE$)). If published, this will include your full peer review and any attached files.


**Do you want your identity to be public for this peer review?** If you choose “no”, your identity will remain anonymous but your review may still be made public.


For information about this choice, including consent withdrawal, please see our [Privacy Policy](https://urldefense.com/v3/__https:/www.plos.org/privacy-policy__;!!H8mHWRdzp34!42p3eJreM2notPrH6CjlnHgQEAEhFoCG9V_ign8GyuIguhaPdFIPNYBp1_Ns8kQorj4pxQ622S3ucqA$).

Reviewer #1: No

Reviewer #2: **Yes:** Selam Degu
